# Vesicocutaneus fistula after cesarean section-a curious complication: Case report and review

**DOI:** 10.4274/tjod.71135

**Published:** 2016-03-10

**Authors:** Burak Tatar, Ebru Erdemoğlu, Sedat Soyupek, Yakup Yalçın, Evrim Erdemoğlu

**Affiliations:** 1 Süleyman Demirel University Faculty of Medicine, Department of Gynecologic and Oncology, Isparta, Turkey; 2 Isparta Maternity Hospital, Clinic of Obstetrics and Gynecology, Isparta, Turkey; 3 Süleyman Demirel University Faculty of Medicine, Department of Urology, Isparta, Turkey

**Keywords:** Cesarean delivery, vesicocutaneous fistula, urachus, diagnosis, treatment

## Abstract

Vesicocutaneous fistulas are very rare pathologies in the urinary tract. We present the second case of a vesicocutaneus fistula after cesarean section, and discuss strategies for prevention, diagnosis, and treatment of this exceptional complication. A woman with a vesicocutaneous fistula after cesarean delivery was admitted and diagnostic tests including fluoroscopy, magnetic resonance imaging (MRI), and reconstructed MRI revealed the fistula tract and an urachal anomaly. The patient was treated through excision of the fistula tract. Laparotomy should be performed carefully, and the surgeon should be aware of the urachus. Inadvertent trauma to the urachus during laparotomy might cause serious unexpected complications. Possible etiologic factors for vesicocutaneous fistulae, prevention, and treatment methods are discussed.

## PRECIS:

We present the second reported case of vesicocutaneus fistula occurring after cesarean section, and discuss strategies for prevention, diagnosis, and treatment of this exceptional complication.

## INTRODUCTION

The cesarean delivery rate has risen from 4.5 percent to 31.8 percent of all deliveries^(1)^. Lower urinary tract injuries are not infrequent and may occur during this procedure. If not intraoperatively detected and repaired, diagnosis will be delayed and a genitourinary fistula may be encountered later. There are case reports of vesico-vaginal, uretero-vaginal, vesico-uterine fistulas after cesarean section. There are also reported cases of uretero-vesico-cervical and vesico-peritoneal fistulas^(2,3,4)^. Vesicocutaneous fistulas are very rare pathologies in the urinary tract. Trauma, neoplasia, inflammation, and iatrogenic injury are the most frequent causes. Anomalies of the urachus may predispose patients to injury and subsequent fistula formation during surgery due to the distorted anatomy. We present the second case of vesicocutaneus fistula, which occurred after cesarean section, and discuss strategies for prevention, diagnosis, and treatment of this exceptional complication.

## CASE REPORT

A gravida 2, para 2 woman aged 44 years with a history of two cesarean sections was referred because of recurrent urinary tract infection and urinary leakage through the abdomen. In her past medical history, she had Sjogren’s syndrome and optic neuritis. In her past surgical history, she had a bladder injury in a caesarean section, which was repaired with a primary suture. One month later, she presented with a 10 cm subcutaneous hematoma at its largest dimension. The hematoma was drained surgically, and during the exploration, no source of bleeding was found. Four months later (in total, five months after the cesarean section), she was admitted to the hospital and referred for urinary leakage through the abdomen, about 5 cm below the level of umbilicus. The leakage was periodic and she had an episode of subcutaneous abscess, which was managed conservatively using antibiotics and non-steroidal anti-inflammatory drugs.

**Figure 1:**
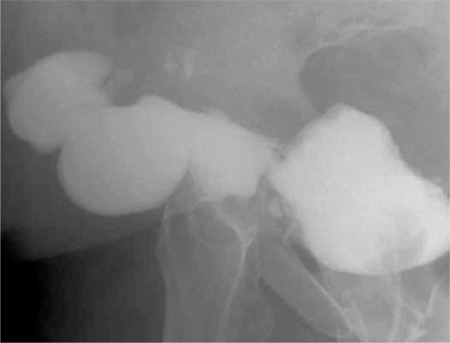
Fluoroscopic lateral view

**Figure 2:**
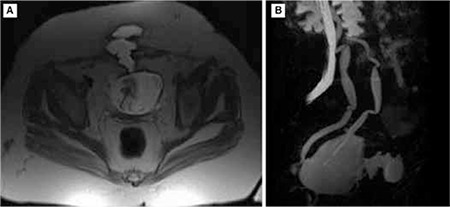
A: T2 Weighted image of the vesico-cutaneous fistula. B: Reconstructed lateral-oblique magnetic resonance image. Fistula tract is clearly seen

 A fistula tract was observed in a retrograde fluoroscopic examination; however, the distal end of the fistulous tract was not clear (Figure 1). An ultrasound examination was also not helpful to clarify the lesion. Contrast-enhanced magnetic resonance (MR) urography (Figure 2) revealed a defect of 1.2 cm on the anterior wall of the bladder in the midline. Between this defect and subcutaneous fatty tissue, a fistula tract of 2 cm at its widest diameter was evident. In the subcutaneous fatty tissue, a 6x6.4x3.7 cm collection was directly related with the fistula tract. There was inflammation and possible infection surrounding the collection due to the dense contrast enhancement.

**Figure 3:**
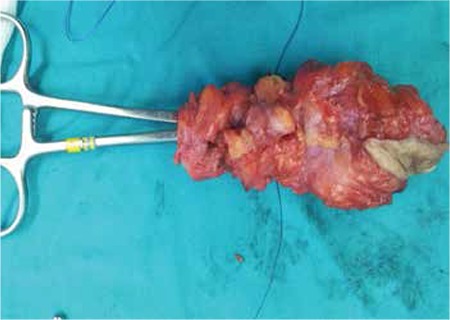
Fistula tract totally excised with the outer edge on the skin

**Figure 4:**
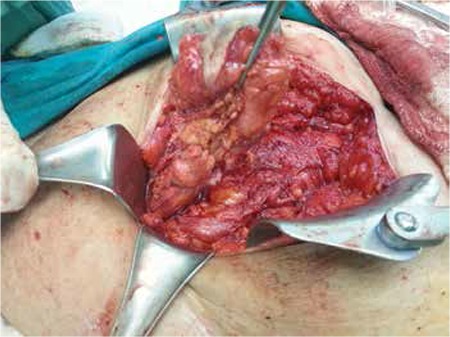
Excision of the fistula tract via laparotomy

The patient underwent a laparotomy with the plan for excision of the fistula tract and repairing the bladder defect. A suprapubic incision parallel to the Langer’s lines of the skin was made with a 1.5-2 cm margin with the fistulous tract. The fistula tract was about 12 cm in length and 4 cm wide, and was totally excised with the involved skin (Figure 3, 4). The defect on the dome of the bladder, which is apart from the trigon, was closed in 3 layers. The rectus fascia was closed with 1-0 Vicryl Subcuticular tissue was repaired with interrupted 2-0 Vicryl and the skin was repaired with 3-0 Prolene sutures. Her two-month follow-up after the surgery was unremarkable.

## DISCUSSION

Vesicocutaneus fistulas are rare. Their exact incidence is not known due to their rarity. Trauma, neoplasia, inflammation, and iatrogenic injury are the most frequent causes of these urinary fistulas^(1,5)^. Due to the rarity of vesicocutaneus fistulas, their risk factors are not clearly identified. Pelvic radiation, radical hysterectomies, pelvic fractures, hip arthroplasties, bladder calculus, and inguinoscrotal hernia operations are reported risk factors^(6)^. Actinomyces infections and factitious disorders have also been reported to cause vesicocutaneous fistula^(7,8)^. Unrecognized urachal anomalies may be a predisposing risk factor during surgical procedures. Cesarean delivery might be a risk factor due to the nature of the procedure, compared with vaginal delivery. There was an urachal anomaly, a possible urachal diverticula that was asymptomatic until the surgical intervention in our case. Urachal anomalies are very rare. Their true incidence is unknown, but is estimated to be between 0.015 to 0.13 in 1000 births according to a large case series^(6,7)^. They may be asymptomatic, especially when presenting as a urachal cyst or diverticulum. These anomalies may predispose a patient to injury during abdominal surgery, especially in cesarean section. Inadvertent injury to the urachus with a diverticulum may be complicated by vesicocutaneous fistula.

Radiographic imaging is the traditional method to identify a fistula. Fluoroscopy may identify the lesion, but it may not clarify it. Sonography was one of the imaging modalities used in the past, but due to its poor sensitivity, especially in identifying complexity, size, and multiplicity, it is rarely used now for identification of fistulae^(8)^. Magnetic resonance imaging and computed tomography (CT) are currently the imaging modalities of choice for the initial evaluation of patients with suspected pelvic fistulae^(9)^. MR seems to be one of the most sensitive methods to define the exact location and complexity of a fistula tract, with more than 90% sensitivity and correlates well with surgical findings^(10)^. Multi-detector CT is the choice for patients who are unable to tolerate fluoroscopy or MR imaging. As in our case, the best imaging modality was contrast-enhanced magnetic resonance (MR) imaging, which clearly identified the exact location of the lesion and its relation with the surrounding tissues.

There is one case report of vesicocutaneus fistula secondary to cesarean hysterectomy from Pakistan by Toufique and Merani^(11)^. It was treated with conservative management and an indwelling bladder catheter was left for 3 weeks and parenteral antibiotics were given. The authors reported that the fistula tract closed spontaneously. A conservative approach may be tried in small fresh fistulas. We chose a curative surgical approach for a major fistula. The treatment approach depends on type of the fistula, symptoms, the time passed from injury, and the patient. For vesico-vaginal fistula, fistulectomy is the preferred management method. Delayed repair is the classic approach, but some articles advocated immediate repair because of better results^(12,13,14)^. Conservative management is also described, but an option not chosen frequently^(15)^. When the case is a uretero-vaginal fistula, conservative management can be considered initially, especially if the patient is appropriate for ureteral stenting, otherwise surgery is the definitive treatment method^(16)^. Vesicocutaneous fistulas are very rare, and to the best of our knowledge, there has been no report of a vesicocutaneus fistula occurring secondary to cesarean section and as a consequence, the optimal management strategy is unknown.

Although conservative management can be reasonable in asymptomatic patients with urachal remnants, fistulectomy with primary closure of the bladder is a logical approach, especially for symptomatic patients with recurrent infections as in our case^(17)^. Laparotomy should be performed carefully, and the surgeon should be aware of patent urachus. Inadvertent trauma to the urachus during laparotomy might be a cause for serious unexpected complications.
